# Correlation of Osteoporosis in Patients With Newly Diagnosed Type 2 Diabetes: A Retrospective Study in Chinese Population

**DOI:** 10.3389/fendo.2021.531904

**Published:** 2021-05-14

**Authors:** Yuhua Wen, Huijuan Li, Xiaoya Zhang, Peipei Liu, Jing Ma, Liya Zhang, Keqin Zhang, Lige Song

**Affiliations:** ^1^ Department of Endocrinology, Tongji Hospital, Tongji University School of Medicine, Shanghai, China; ^2^ Institute of Osteoporosis and Metabolic Bone Diseases, Tongji University School of Medicine, Shanghai, China

**Keywords:** risk assessment, type 2 diabetes mellitus, TRACP-5b, osteocalcin, osteoporosis, bone specific alkaline phosphatase

## Abstract

This study aimed to explore the risk factors attributed to osteoporosis in newly type 2 diabetes mellitus (T2DM) patients. This study aimed to recruit 244 T2DM patients and 218 non-diabetic controls. We collected demographic characteristics, medical history, bone mineral density and biomarkers including bone specific alkaline phosphatase (BALP), osteocalcin, N-terminal peptide of type I procollagen (P1NP), tartrate-resistant acid phosphatase 5b (TRCAP-5b), β-Cross Laps of type I collagen-containing cross-linked C-telopeptide (β-CTX), 25-hydroxyvitamin D, parathyroid hormone were recorded or detected. Bone mineral density (BMD) was our primary outcome. Based on the result of BMD, we divided both the control group and T2DM group into three subgroups: normal bone mass, osteopenia and osteoporosis. In control group, we found age, sex, menopausal status, BMI, P1NP, BALP, TRACP-5b, osteocalcin, and corrected serum calcium are differential among three subgroups. In T2DM group, we found age, sex, menopausal status, drinking status, BMI, HbA1c, TRACP-5b and OC were differential among three subgroups. In T2DM and control groups, age, female, postmenopausal status, BALP, TRACP-5b and osteocalcin were positively correlated while BMI was negatively correlated with osteoporosis. In control group, β-CTX was positively correlated with osteoporosis. In T2DM group, HbA1c and corrected serum calcium concentration were positively correlated with osteoporosis. After further adjustment of age, BMI in male, TRACP-5b was positively correlated with the risk of osteoporosis in newly diagnosed T2DM. After adjusted of age, BMI and menopausal status in female, OC was positively correlated with the risk of osteoporosis in newly diagnosed T2DM and controls. In female T2DM, BALP and P1NP were positively correlated with the risk of osteoporosis. In conclusion, age, BMI and menopausal status are common risk factors for osteoporosis in diabetic and non-diabetic patients, however TRACP-5b, BALP and osteocalcin are special risk factors for osteoporosis in newly diagnosed T2DM patients but not non-diabetic patients, which may be applied to identify osteoporosis risk in T2DM patients, but this result needs to be proven with fracture data.

## Introduction

Type 2 diabetes has affected more than 451 million individuals worldwide, and its prevalence is steadily rising following the aging of the society (1). It induces high mortality mainly due to its chronic complications, like diabetic nephropathy, retinopathy, neuropathy and cardiovascular diseases (2). Recent evidence suggests that skeleton fracture might be considered as one more chronic complication of type 2 diabetes mellitus (T2DM) (3). Fracture risk is rising following the duration of T2DM, including any fractures, spine fractures and hip fractures, but not forearm fractures (4).

Comparing to high fracture risk in type 2 diabetic patients, area bone mineral density (aBMD) based on dual energy X-ray absorptiometry (DEXA) is not differential to non-diabetes controls. Thus, fracture risk prediction based on aBMD underestimates the fracture risk of the patients with T2DM (3). Lots of previous studies have reported that for a given T score, T2DM patients had a higher fracture risk than those without T2DM (5). The fracture risk assessment tool (FRAX) was developed to evaluate the 10-year probability of hip fracture and major osteoporotic fracture by the World Health Organization. However, FRAX also underestimates the fracture risk of the type 2 diabetic patients even after it included femoral neck aBMD and several clinical variables (age, sex, body mass index, use of glucocorticoids, current smoking, alcohol intake of three or more units per day, secondary osteoporosis, rheumatoid arthritis, prior fragility fracture) (5). Because of the widespread use of bone mineral density (BMD) and FRAX for predicting fracture risk in populations, lots of efforts have been put to explore the more precise way to use BMD and FRAX on fracture risk prediction in type 2 diabetic patients. The results showed that type 2 diabetic patients with femoral neck BMD T score at −2.1 in men and −1.9 in women have the same fracture risk in the non-diabetic populations with femoral neck BMD T score at −2.5 (5). If T2DM replaces RA in FRAX for evaluating fracture risk of the T2DM patients, this tool becomes more precise (6). These above results imply that T2DM patients have unique bone metabolism characteristics and risk factors that contribute to their higher fracture risk.

Many studies have been conducted to explore the mechanisms of fracture risk in type 2 diabetic patients (7). Results showed that increased risk of falls induced by hypoglycemic events (8), poor muscle strength (9), poor vision (caused by diabetic retinopathy) (10), and peripheral neuropathy (11) contributes to large amount of fractures in type 2 diabetic patients. Impaired renal function in long duration diabetic patients might interfere with vitamin D metabolism, which results in reduced muscle strength and impaired bone metabolism (11). Some studies find that impaired bone strength induced by bone materials imbalance and bone microstructure deterioration attributes to fracture risk in type 2 diabetic patients (12, 13). Moreover, some anti-diabetic drugs including TZDs, SGLT-2 inhibitors and insulin can increase fracture risk in the T2DM population (14).

However, no previous study has been conducted to explore the change of bone mineral density and bone turnover in newly diagnosed type 2 diabetic patients who are not companied with chronic diabetic complications and untreated. Thus, this study may uncover or hint unique risk factors or mechanisms of fracture risk in type 2 diabetic patients.

## Materials and Methods

### Study Design

This was a retrospective study. Tongji Hospital of Tongji University School of Medicine is one of the large-scale general hospitals in Shanghai, China, which integrates medical service, education, and research. Participants were recruited from all the patients who attend the Endocrine outpatient clinic from January 2014 to February 2018.

### Participants

812 individuals with newly-onset type 2 diabetes and 759 individuals without diabetes, who do not meet the exclusion criteria, were included in this study. Exclusion criteria for patient selection were as follows: 1) the history of fragility fractures (fractures influence bone turnover markers in the 6 months after fractures happen); 2) with secondary osteoporosis; 3) exposed to medicine associated with osteoporosis; 4) with malignant tumors; 5) with autoimmune diseases; 6) treated by antidiabetic drugs.

244 newly diagnosed type 2 diabetes patients and 218 non-diabetic individuals were enrolled. Clinical characteristics, fracture history, whole blood cell analysis, biochemical indicator, electrolytes, bone turnover markers were recorded at the first visit and bone mineral density (BMD) was assessed three-month after the first visit. Statistical analysis was used to explore the risk factors attributed to osteoporosis in newly type 2 diabetic patients.

The diagnosis of type 2 diabetes depends on the criteria of WHO (1999) (15). The individuals who were included in this study should also have been tested for liver function, renal function, fasting plasma glucose (FPG), glycosylated hemoglobin (hemoglobin A1c, HbA1c), fasting C-peptide (CPS), serum electrolyte indices, thyroid function, calcium (Ca), phosphorus (P), parathyroid hormone (PTH), 25-hydroxyvitamin D (25(OH)D) and bone turnover markers (N-terminal peptide of type I procollagen (P1NP), β-Cross Laps of type I collagen-containing cross-linked C-telopeptide (β-CTX), bone specific alkaline phosphatase (BALP), tartrate-resistant acid phosphatase 5b(TRACP-5b), osteocalcin (OC) at the first time of clinical visit. Bone mineral density (BMD) at three sites (lumbar, femur neck, and total hip) were detected by dual-energy X-ray absorptiometry (DXA) scans 3 months after the first time of clinical visit.

After applying these exclusion and inclusion criteria, 244 newly diagnosed type 2 diabetes mellitus (DM) and 218 non-diabetic mellitus (Non-DM) individuals were included in this study. The Ethics Committee approved this study of Tongji Hospital of Tongji University School of Medicine and all the participants signed the informed consents. Based on the BMD from DEXA, we divided participants into three subgroups: normal, osteopenia, and osteoporosis according to WHO osteoporosis diagnostic criteria (16). Briefly, the lowest T scores in the three sites were used to classify the individuals into three subgroups: normal (T score >−1), osteopenia (T score between −1 and −2.5), and osteoporosis (T score <−2.5).

### Data Collection

The general characteristics of the enrolled individuals were recorded during the first time of clinical visit, including age, gender, menopause status, drinking, smoking, body weight, and height. The smoking status depends on whether the individual is currently smoking. The alcohol consumption depended on whether the patient takes three or more units of alcohol daily (one unit of alcohol means 8 g of alcohol). Liver function, renal function, FPG, and electrolytes were detected by Advia-1650 automatic chemistry analyzer (Bayer Diagnostics, Tarletown, NY, USA). CPS was measured by chemical luminescence (Siemens CENTAUR XP Chemical Glow Immunoassay Analyzer). P1NP, OC, β-CTX, 25(OH)D, PTH were measured by electrochemiluminescence assay (Roche Diagnostics). BALP and TRACP-5b were measured by enzyme immunoassay (IDS Ltd). HbA1c was measured by high-performance liquid chromatography. Corrected Ca(mmol/L) was calculated as the formula: Ca(mmol/L) + 0.02 × [40-albumin(g/L)]. Bone mineral density (BMD) at lumbar, femur neck, and total hip were detected by dual-energy X-ray absorptiometry (DXA) scans (DXA, HOLOGIC Discovery; coefficient of variation <1%).

### Statistical Analysis

The percentage was used to describe the categorical variables. Continuous variables in normal distribution were expressed as mean ± SD. Median and percentages were used for continuous variables that were not in the normal distribution. Chi-squared tests analyzed comparisons between the categorical variables. The analysis of variance (ANOVA) was applied in continuous variables that were in the normal distribution. Kruskal–Wallis test was used in continuous variables that were not in the normal distribution. The logistic regression analysis without adjustment was performed including potential indicators. Then the data has stratified the cohorts by gender. In the male model, we adjusted BMI and age. And, in the female model, we adjusted age, menopause status, and BMI. A P-value <0.05 was considered statistically significant. All statistical calculations were performed using the Statistical Package for the Social Sciences software for Windows version 20.0 (IBM, Armonk, NY, USA).

## Results

Comparing to type 2 diabetic individuals, the proportion of smoking and drinking in non-diabetic individuals was lower (6.9% vs 21.7% and 3.2% vs 10.7%) and their BMI was also lower (25.28 ± 3.87 kg/m^2^ vs 23.39 ± 3.98 kg/m^2^). Serum 25-hydroxyvitamin D level in diabetic patients was lower when compared to the non-diabetic individuals (31.68 vs 38.91 nmol/l) (**Supplementary Table 1**).

In the control group, we found age (P-value <0.001), gender (P-value = 0.011), postmenopausal status (P-value <0.001), BMI(P-value <0.001), correlated Ca (P-value = 0.034), P1NP (P-value = 0.002), BALP (P-value = 0.009), TRACP-5b (P-value = 0.015), OC (P-value <0.001) are differential among three subgroups (**Table 1**). And there is weak or no evidence against the null hypothesis that current drinking, current smoking, FPG, HbA1c, Ca, P, PTH, 25(OH)D were the same in three subgroups. In the type 2 diabetic group, older age, female, postmenopausal status, and lower BMI were related to a higher possibility of osteoporosis. Current smoking had no relationship with the bone mass decline in type 2 diabetic groups, but the rate of current drinking is lower in the osteoporosis patients in type 2 diabetic group but not in the non-diabetic group (**Table 1**).

**Table 1:** Baseline characteristics of patients with T2DM and non-diabetic controls.

Variables	Non-DM				DM			
	Normal (N = 23)	Osteopenia (N = 78)	Osteoporosis (N = 117)	P-value	Normal (N = 67)	Osteopenia (N = 118)	Osteoporosis (N = 59)	P-value
Age (years)	56.44 ± 15.86	64.94 ± 12.48	69.98 ± 12.16	P <0.001	55.00 ± 13.46	59.29 ± 11.14	65.73 ± 10.08	P <0.001
Gender (Male, %)	9 (39.1%)	21 (26.9%)	17 (14.5%)	P = 0.011	37 (55.2%)	71 (60.2%)	21 (35.6%)	P = 0.008
Postmenopausal (%)	9 (39.1%)	51 (65.4%)	98 (83.8%)	P <0.001	22 (32.8%)	44 (37.3%)	37 (62.7%)	P = 0.001
Current smoking (%)	2 (8.7%)	4 (5.1%)	9 (7.7%)	P = 0.736	17 (25.4%)	29 (24.6%)	7 (11.9%)	P = 0.101
Current drinking (%)	0 (0.0%)	2 (2.6%)	5 (4.3%)	P = 0.745	9 (13.4%)	16 (13.6%)	1 (1.7%)	P = 0.038
BMI (kg/m2)	26.34 ± 5.22	23.81 ± 3.36	22.54 ± 3.79	P <0.001	27.22 ± 4.23	25.26 ± 3.49	23.11 ± 2.99	P <0.001
FPG (mmol/L)	4.93 (4.61–5.35)	5.07 (4.72–5.54)	4.95 (4.71–5.39)	P = 0.361	9.39 (7.10–11.40)	9.39 (7.47–12.29)	9.43 (7.86–12.52)	P = 0.448
HbA1c (%)	5.71 ± 0.31	5.77 ± 0.34	5.74 ± 0.36	P = 0.680	10.27 ± 2.54	10.78 ± 2.47	11.58 ± 2.10	P = 0.010
Ca (mmol/L)	2.20 ± 0.11	2.21 ± 0.11	2.23 ± 0.14	P = 0.491	2.22 ± 0.10	2.21 ± 0.11	2.23 ± 0.14	P = 0.454
Correlated Ca (mmol/L)	2.22 (2.14–2.28)	2.23 (2.17–2.28)	2.25 (2.21–2.31)	P = 0.034	2.23 (2.17–2.28)	2.22 (2.18–2.28)	2.25 (2.20–2.30)	P = 0.125
P (mmol/L)	1.21 ± 0.22	1.19 ± 0.17	1.23 ± 0.18	P = 0.355	1.24 ± 0.16	1.21 ± 0.20	1.21 ± 0.23	P = 0.561
PTH (pg/ml)	46.34 (39.2–60.11)	44.75 (32.85–57.11)	45.3 (36.41–56.51)	P = 0.583	39.00 (31.78–49.14)	39.00 (32.47–48.44)	39.00 (29.40–48.87)	P = 0.574
25(OH)D (nmol/L)	41.46 (22.87–55.63)	38.9 (28.65–45.27)	37.91 (27.58–48.67)	P = 0.591	33.73 (25.43–42.78)	31.68 (24.82–43.62)	30.20 (23.67–39.24)	P = 0.239
P1NP (ng/mL)	41.00 (27.10–48.10)	45.55 (33.53–61.43)	55.40 (40.30–68.12)	P = 0.002	38.20 (32.50–49.41)	38.20 (30.54–47.22)	38.20 (33.40–56.10)	P = 0.269
CTX (ng/mL)	0.36 (0.25–0.56)	0.45 (0.23–0.58)	0.49 (0.29–0.69)	P = 0.072	0.38 (0.24–0.55)	0.38 (0.27–0.47)	0.44 (0.32–0.71)	P = 0.056
BALP (μg/L)	11.80 (9.40–15.10)	14.73 (11.13–20.15)	15.40 (12.65–20.10)	P = 0.009	18.10 (13.50–20.20)	18.30 (15.35–22.13)	18.30 (15.70–26.20)	P = 0.083
TRACP-5b (U/L)	1.82 ± 0.52	2.26 ± 0.72	2.28 ± 0.73	P = 0.015	2.18 ± 0.61	2.32 ± 0.59	2.57 ± 0.74	P = 0.003
OC (ng/mL)	15.26 (11.84–19.68)	19.20 (14.55–22.93)	21.68 (15.68–29.27)	P <0.001	12.83 (11.01–15.44)	12.83 (10.08–14.85)	15.19 (12.71–19.57)	P = 0.001

*P < 0.05. Values are shown as median (p25–p75), means ± SD or number (percentage).

**Figure 1:**
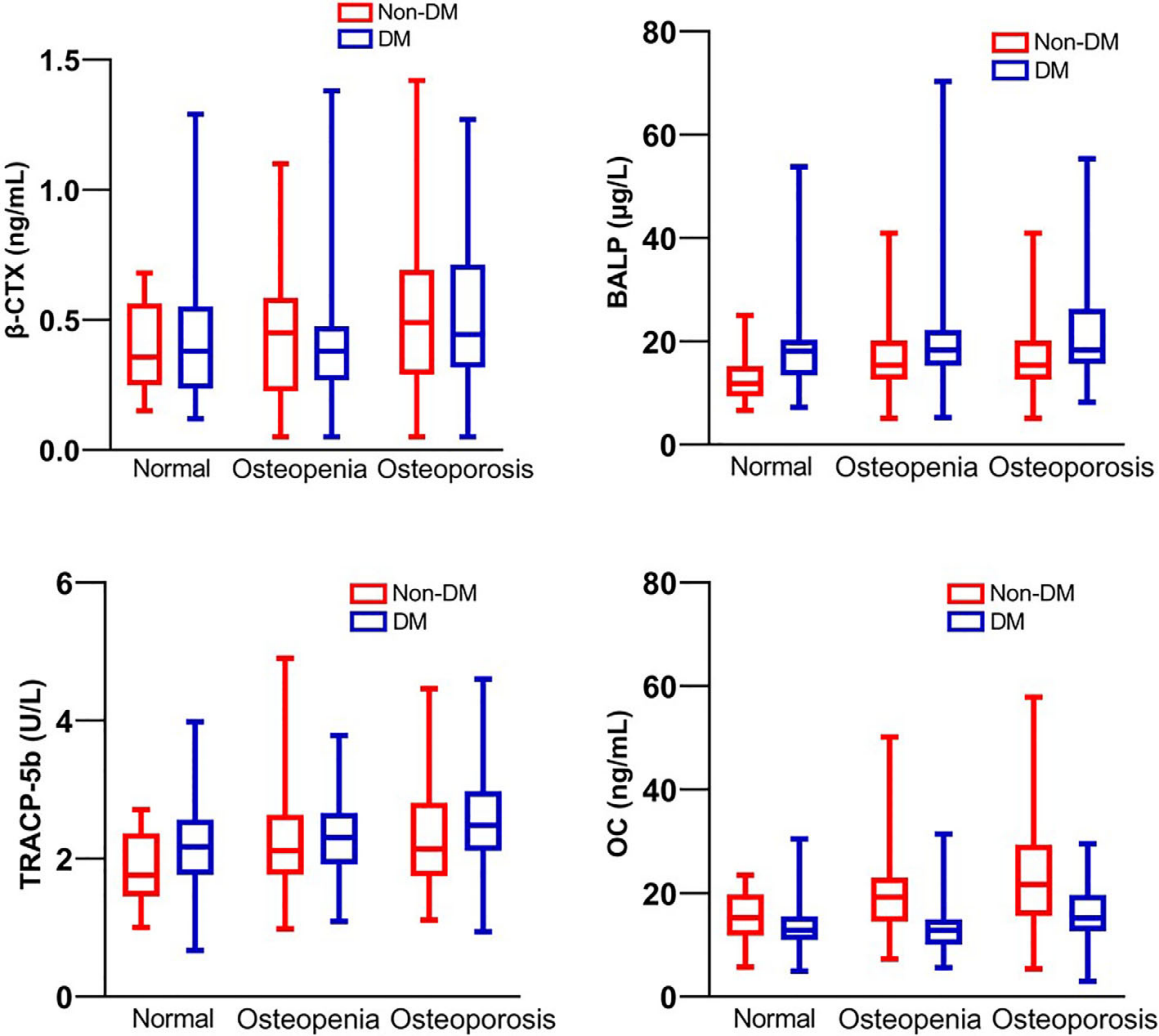
Bone turnover markers β-CTX, BALP, TRACP-5b and OC of patients with T2DM and non-diabetic controls.

According to glucose level, FPG had no difference between normal, osteopenia, and osteoporosis subgroups in both non-diabetic and type 2 diabetic groups, but higher HbA1c was related to lower BMD in the type 2 diabetic group (**Table 1**). Total calcium level (normalized by serum albumin concentration) is positively related to the bone mass decline in the non-diabetic group but not in the diabetic group, however, the level of calcium-regulating hormones, PTH and 25(OH)D, had no difference between normal, osteopenia and osteoporosis subgroups in both type 2 diabetic and non-diabetic groups (**Table 1**). According to bone turnover markers, levels of TRACP-5b and OC were higher in the osteoporotic group comparing to osteopenia and normal groups in both type 2 diabetic and non-diabetic populations, and the levels of P1NP and BALP had the same trend in the non-diabetic group but not in the diabetic group. β-CTX level showed no difference between the three subgroups in both diabetic and non-diabetic populations (**Table 1** and **Figure 1**).

In the control group, logistic regression analysis showed that age, postmenopausal status, correlated Ca, β-CTX, BALP TRACP-5b, and OC could increase the risk of osteoporosis while BMI could decrease the risk (**Table 2**). Interestingly, current drinking was negatively related to the risk of osteoporosis in the diabetic group but not in the non-diabetic group (**Table 2**). In diabetic groups, HbA1c, BALP, TRACP-5b, and OC levels could increase the risk of osteoporosis (P <0.05). P1NP neither increases nor decreases the risk of osteoporosis in diabetic and non-diabetic groups (**Table 2**).

**Table 2:** The relationship between the indicators with the risk of osteoporosis in diabetes and non-diabetics.

Variables	Non-DM			DM		
	Odds ratio	95% CI	P-value	Odds ratio	95% CI	P-value
Age (years)	1.050	1.028–1.072	<0.001*	1.053	1.032–1.076	<0.001*
Gender (Male, %)	0.395	0.213–0.734	0.003*	0.659	0.487–0.892	0.007*
Postmenopausal (%)	3.877	2.150–6.991	<0.001*	2.292	1.404–3.743	0.001*
Current drinking (%)	2.374	0.445–12.660	0.311	0.519	0.325–0.828	0.006*
BMI (kg/m^2^)	0.862	0.805–0.924	<0.001*	0.813	0.758–0.871	<0.001*
HbA1c (%)	0.925	0.442–1.937	0.837	1.160	1.051–1.281	0.003*
Correlated Ca(mmol/L)	3.914	0.943–16.248	0.06	12.587	1.024–154.788	0.048*
PTH (pg/ml)	1.004	0.992–1.017	0.51	0.996	0.982–1.011	0.582
25(OH)D (nmol/L)	1.003	0.988–1.018	0.705	0.993	0.977–1.009	0.381
P1NP (ng/ml)	1.003	0.995–1.011	0.397	1.011	0.997–1.025	0.121
β-CTX (ng/ml)	3.721	1.297–10.674	0.015*	2.045	0.776–5.384	0.148
BALP (μg/L)	1.021	1.001–1.042	0.043*	1.031	1.003–1.060	0.029*
TRACP-5b (U/L)	1.324	1.072–1.635	0.009*	1.932	1.324–2.819	0.001*
OC (ng/ml)	1.040	1.021–1.059	<0.001*	1.066	1.016–1.119	0.009*

*P < 0.05. Logistic regression analysis without adjustment.

To clarify the relationship between different bone turnover markers and HbA1c with the occurrence of osteoporosis, we built four models: male with T2DM, male without T2DM, female with T2DM, and female without T2DM. We adjusted age and BMI in males with T2DM, males without T2DM model, and adjusted age, BMI, and menopausal status in the female with T2DM and female without T2DM. For males, the results showed TRACP-5b and osteoporosis are potential risk factors of osteoporosis in the diabetic model, but not in the non-diabetic model. For women, we found the OC level was a potential risk factor for osteoporosis in the diabetic and non-diabetic models. P1NP and BALP were potential risk factors of osteoporosis only in the female with T2DM model but not in female without T2DM model (**Table 3**).

**Table 3:** The relationship between the indicators with the risk of osteoporosis in diabetes and non-diabetics with gender stratification and adjustment.

Variables	Male						Female					
	Non-DM			DM			Non-DM			DM		
	Odds ratio	95% CI	P-value	Odds ratio	95% CI	P-value	Odds ratio	95% CI	P-value	Odds ratio	95% CI	P-value
Current drinking (%)	5.855	0.886–38.68	0.067	0.435	0.182–1.041	0.061						
HbA1c (%)	0.509	0.111–2.347	0.387	1.085	0.943–1.249	0.255	1.442	0.519–4.004	0.482	1.097	0.933–1.29	0.262
Correlated Ca (mmol/L)	4.92	0.351–68.937	0.237	6.773	0.119–385.677	0.354	3.038	0.545–16.942	0.205	15.066	0.489–464.619	0.121
PTH (pg/ml)	0.991	0.953–1.031	0.651	0.985	0.963–1.009	0.22	1.005	0.99–1.02	0.511	1.006	0.984–1.028	0.609
25(OH)D (nmol/L)	0.963	0.924–1.005	0.082	0.99	0.968–1.013	0.376	1.013	0.993–1.033	0.203	0.989	0.961–1.018	0.456
P1NP (ng/ml)	1.025	0.992–1.06	0.138	1.009	0.984–1.035	0.47	0.999	0.992–1.005	0.688	1.026	1.005–1.047	0.014*
β-CTX (ng/ml)	0.412	0.02–8.343	0.563	2.383	0.445–12.756	0.31	3.177	0.869–11.611	0.081	1.791	0.45–7.117	0.408
BALP (μg/L)	1.03	0.957–1.109	0.426	1.02	0.972–1.071	0.419	1.049	1–1.1	0.052	1.053	1.013–1.095	0.009*
TRACP-5b (U/L)	2.095	0.917–4.787	0.079	2.204	1.215–3.995	0.009*	1.161	0.734–1.838	0.524	1.446	0.829–2.522	0.194
OC (ng/ml)	1.079	0.985–1.184	0.103	1.018	0.941–1.103	0.65	1.058	1.016–1.101	0.006*	1.113	1.034–1.198	0.004*

*P < 0.05. Logistic regression analysis with adjustment of age and BMI in male and adjustment of age, BMI, and menopausal status in female.

## Discussion

Age and menopause are the major determinants for the risk of osteoporosis and its related fragile fractures. In the Chinese population, recently published data from a multicenter, large-scale study showed that osteoporosis increases prominently as age increases in both men and women after 55 based on BMD of lumbar spine femoral neck total femur detected by DEXA (17). In worldwide, the number of estimated individuals with osteoporosis and its related fractures by 2020 will steadily increase following the aging of the whole society. More and more efforts have been put into preventing osteoporotic fractures (18). The prevalence of osteoporosis in men (4.3%) is lower than in women (15.4%) according to estimates of the number of US residents in 2010 (19). For women, after menopause, their bone mass decreases about 12–20% in the first five years (20). Thus, lower peak bone mass and postmenopausal estrogen deficiency induce higher fracture risk and earlier occurrence of fragile fractures in women comparing to men (21). Data have shown that compared with men, osteoporosis is four times higher in women aged 50 years and older, and the incidence of osteopenia is two times higher (22, 23). The result in **Table 2** showed that the current drinking was negatively related to the risk of osteoporosis in the diabetic group. The result in **Table 3** showed that after adjusted by age, BMI, and menopausal status, there is weak evidence against current drinking is neither a risk factor nor a protective factor. This result contradicts to some early studies. We believe the explanation is patients who take alcohol are more likely to have higher BMI. According to some studies, low BMI is an indicator for osteoporosis and its related fractures, and the study conducted in United Stated has shown that BMI (<28 kg/m^2^) in White women should be put into a priority for BMD screening (24). The popularity of BMI on evaluating fracture risk base on its easy way to measure, but it cannot distinguish between fat mass and lean mass. Studies have shown that fat mass is inversely, but lean mass is positively related to bone mass (25), thus BMI is an important indicator found in other studies.

Type 2 diabetes has been recognized as an independent risk for fragile fractures (5, 26). The high fracture risk in T2DM patients can be induced by hypoglycemia, muscle weakness and the chronic complications (such as retinopathy, neuropathy, and neuropathy) which usually happen in the patient with longer duration of T2DM (27). However, hyperglycemia should always be kept in mind because it plays a vital role during the impaired bone metabolism in T2DM patients, leading to reduced bone strength (7). In this study, the risk of osteoporosis increased following HbA1c level in newly diagnosed T2DM, and this result implies elevated glucose level can damage bone metabolism even in the early stage of T2DM. Hyperglycemia and its associated hyperosmolarity also suppress the expression of genes associated with osteoblast maturation (28). Hyperglycemia causes calcium homeostasis imbalance by inhibiting the bone formation and accelerating bone resorption. The clinical manifestation is a decrease in primary and secondary sponge trabecular bone mass. Increased osteoblast apoptosis induced by high glucose has been demonstrated. Hyperglycemia may inhibit osteogenic transdifferentiation and bone resorption-related gene matrices, including metalloproteinase (MMP) 9 and carbonic anhydrase II (CAII), increased expression levels. At the same time, osteogenesis-related genes Runx2 and alkaline phosphatase (ALP) expression levels decreased (29). Osteogenesis was inhibited when the mesenchymal progenitor cells were exposed to high glucose by activating the Notch2 signaling pathway (30).

AGEs, imbalanced inflammatory cytokines, impaired incretin system, and bone marrow adiposity can also influence the bone metabolism of the T2DM patients. Advanced glycation end products (AGEs) are important causes of inflammatory events that trigger diabetes and its complications (31). Our study found the TRACP-5b is significantly associated with osteoporosis in DM patients while it is not in healthy cohorts. In previous research, the numbers and capacity of osteoclasts and the levels of TRACP-5b were substantially increased in streptozotocin-induced diabetic rats compared with controls (32). The results of the cell experiment used osteoblast-like cells presented evidence that advanced glycation end-products could increase cellular alkaline phosphatase activity at first and regulate osteoblast proliferation and differentiation (33). In primary human osteoblast cultures and osteocyte-like MLO-Y4-A2 cells, by activating the AGE-RAGE pathway (RAGE mRNA up-regulation), HG and AGEs can suppress bone formation by inducing enhanced sclerostin expression in osteocytes. Moreover, AGEs suppressed bone resorption by decreasing RANKL expression and enhanced osteoclastogenesis. Besides, AGEs may cause bone deterioration by impaired matrix mineralization (down-regulation of alkaline phosphatase and osteocalcin mRNA) (34, 35). In mouse stromal ST2 cells, the findings indicated that AGE2 and AGE3 partially inhibited the osteoblastic differentiation of stromal cells by binding to RAGE and decreasing osterix expression. The AGEs not only inhibited cell growth but also increased cell apoptosis. They also suggest that AGE3 increasing TGF-beta expression and secretion, as in diabetes-related bone disorder, the TGF-beta harms bone quality (36, 37).

In short, the accumulation of AGEs and these processes may cause bone matrix damage, bone strength impairment, and low bone turnover in DM (38). Low levels of inflammatory cytokines have a specific protective effect on islet β cells, and the level of inflammatory cytokines is a risk factor for osteoporosis in diabetic patients (39). Generally, IL-1 and tumor necrosis factor-α (TNF-α) not only induce the expression of RANKL to promote the osteoclastogenic process, but they also affect osteoclast activity by executing an anti-apoptotic effect (40). It was reported that serum levels of CRP, as a vital inflammation marker, could independently predict low levels of BMD (41). Low concentration of TNF-α or suppression of TNF-α by TNF soluble binding protein or an anti-TNF-α seems to have a role in suppressing bone resorption by decreasing osteoclast formation induced by the RANKL (42). The incretin system includes glucagon-like peptide-1 (GLP-1) and glucose-dependent insulinotropic polypeptide (GIP). Both of them may promote beta-cell proliferation, prevent apoptosis, and are essential for blood glucose regulation (43). GIP can affect bone health by stimulating osteoblast proliferation and enhancing the expression of collagen type I and the activity of alkaline phosphatase (44). GIP can affect bone health through stimulating osteoblast proliferation, as well as enhancing the expression of collagen type I and the activity of alkaline phosphatase.

Additionally, GIP restrains osteoclast activity by cyclic adenosine monophosphate (cAMP) (45). The GLP-1 may have a positive link with bone strength, as the studies indicated that it promoted osteogenic differentiation (46). In terms of the correlation between bone marrow adiposity and bone health, it has been reported that there is a strong linkage between them. Furthermore, some previous studies prove that bone marrow fat fraction (BMFF) had a negative link with bone mineral density (47). Sheu suggested that compared to the 118 participants without diabetes, 38 older diabetic participants’ vertebral marrow adiposity was higher in the Osteoporotic Fractures in Men (MrOS) study (48). Besides, plenty of studies indicated that marrow adiposity is higher in osteopenic compared to healthy, and in osteoporotic compared to osteopenic people (49).

We are aware of the limitations of our study. The first limitation was that we did not collect the abdominal circumference and hip circumference data, which are important for assessing the relationship between obesity and osteopenia in T2DM. The second limitation was that our study was limited to the Chinese population. There may be differences in bone metabolism between eastern and western populations. The predicted fracture probability of women in mainland China is much lower than that of the United Kingdom when the10-year risk of hip fracture was estimated (50). Another result that needed to be cautious was that we did not find the difference between β-CTX and osteoporosis in different groups. We have known that β-CTX is the most sensitive and stable bone turnover marker. It may cause by the different duration of diabetes or diabetes drugs. We think β-CTX can predict fracture in our follow-up prospective study.

In summary, age, BMI, and menopausal status are common risk factors for osteoporosis in diabetic and non-diabetic patients. However, TRACP-5b, BALP, and osteocalcin are particular risk factors for osteoporosis in newly diagnosed T2DM patients but not non-diabetic patients. The conclusion of this study may be applied to identify osteoporosis risk in Chinese T2DM patients, but this result needs to be proven with a larger population and fracture data.

## Data Availability Statement

The original contributions presented in the study are included in the article/**Supplementary Material**. Further inquiries can be directed to the corresponding author.

## Ethics Statement

This study has been approved by the Ethics Committee of Tongji Hospital, Tongji University School of Medicine (KYSB-2018-136).

## Author Contributions

Conception and design: YW, HL, KZ, and LS. Administrative support: KZ and LS. Provision of study materials or patients: YW, HL, XZ, PL, JM, LZ, KZ, and LS. Collection and assembly of data: YW, HL, XZ, PL, JM, LZ, KZ, and LS. Data analysis and interpretation: YW, HL, XZ, PL, JM, LZ, KZ, and LS. Manuscript writing: all authors. All authors contributed to the article and approved the submitted version.

## Funding

Project supported by Clinical Research Project of Tongji Hospital of Tongji University (Grant No. ITJ(ZD)1904), Shanghai Municipal Health Commission to LS (201840217), Clinical Science and Technology Innovation Program of Hospital Development Center to LS (SHDC12018X10) and Shanghai Science and Technology Foundation to LS (19ZR1448600).

## Conflict of Interest

The authors declare that the research was conducted in the absence of any commercial or financial relationships that could be construed as a potential conflict of interest.
