# Supplementary material for: Correlation of Osteoporosis in Patients With Newly Diagnosed Type 2 Diabetes: A Retrospective Study in Chinese Population
Source: Front Endocrinol (Lausanne). 2021 May 14;12:531904. doi: 10.3389/fendo.2021.531904 (PMC8160464; doi:10.3389/fendo.2021.531904)
Supplement: Supplementary file 1 [file Table_1.docx]

Supplement Table 1. Baseline characteristics of total patients

|  | Non-DM | DM |
| --- | --- | --- |
| variables | Total (N=218) | Total (N=244) |
| Age (years) | 66.75±13.34 | 59.67±12.17 |
| Gender (Male, %) | 47(21.6%) | 129(52.9%) |
| Postmenopausal (%) | 158 (72.5%) | 103(42.2%) |
| Current smoking (%) | 15 (6.9%) | 53(21.7%) |
| Current drinking (%) | 7 (3.2%) | 26(10.7%) |
| BMI (kg/m2) | 23.39±3.98 | 25.28±3.87 |
| FPG (mmol/L) | 4.98(4.70-5.42) | 9.39(7.40-12.19) |
| HbA1c (%) | 5.75±0.35 | 10.83±2.44 |
| Ca (mmol/L) | 2.22±0.12 | 2.21±0.12 |
| Correlated Ca (mmol/L) | 2.24(2.19-2.29) | 2.23(2.18-2.29) |
| P (mmol/L) | 1.21±0.18 | 1.22±0.20 |
| PTH (pg/mL) | 45.30(35.47-57.11) | 39.00(31.70-48.72) |
| 25(OH)D (nmol/L) | 38.91(27.98-48.53) | 31.68(25.16-42.49) |
| P1NP (ng/mL) | 49.00(35.45-64.33) | 38.20(31.83-51.11) |
| CTX (ng/mL) | 0.46(0.27-0.65) | 0.38(0.27-0.53) |
| BALP (μg/L) | 14.75(11.50-19.30) | 18.30(15.00-22.23) |
| TRACP-5b (U/L) | 2.23±0.72 | 2.34±0.65 |
| OC (ng/mL) | 19.68(14.44-24.77) | 12.83 (10.92-16.13) |
